# A generic workflow for effective sampling of environmental vouchers with UUID assignment and image processing

**DOI:** 10.1093/database/bax096

**Published:** 2018-01-09

**Authors:** Dagmar Triebel, Wolfgang Reichert, Simone Bosert, Martin Feulner, Daniel Osieko Okach, Abderachid Slimani, Gerhard Rambold

**Affiliations:** 1 Staatliche Naturwissenschaftliche Sammlungen Bayerns, 80638 München, Germany; 2 University of Bayreuth, 95440 Bayreuth, Germany; 3 JOOUST, 40601 Bondo, Kenya; 4 BADJI Mokhtar University, 23000 Annaba, Algeria

## Abstract

Sampling of biological and environmental vouchers in the field is rather challenging, particularly under adverse habitat conditions and when various activities need to be handled simultaneously. The workflow described here includes five procedural steps, which result in professional sampling and the generation of universally identifiable data. In preparation for the field campaign, sample containers need to be labelled with universally unique identifier (UUID)-QR-codes. At the collection site, labelled containers, sampled material and attached supplementary information are imaged using a GNSS- respectively GPS-enabled smartphone or camera. Image processing, tagging and data storage as CSV text file is subsequently achieved in a field station or laboratory. For this purposes, the newly implemented tool *DiversityImageInspector* (URL: http://diversityworkbench.net/Portal/DiversityImageInspector) is used*.* It addresses combined image and data processing in such a context including the extraction of the QR-coded UUID from the image content and the extraction of geodata and time information from the Exif image header. The import of the resulting data files into a relational database or other kind of data management systems is optional but recommended. If applied, the import might be guided by a data transformation tool with compliant schema as described here. The new approach is discussed also with regard to implications for virtual research environments and data publication networks.

**Database URL**: http://diversityworkbench.net/Portal/DiversityImageInspector

## Introduction

In monitoring and inventory projects, scientific proof of observations of investigated organisms and of physical objects in general is mandatory. In the case of easily identifiable macro-organisms, image, video and audio documentation may be considered as sufficient. However, most life science and environmental research projects require collection of samples of organisms or parts thereof, or of environmental materials like soil or water samples. Such specimens are used as reference or study vouchers being processed in the laboratory. Some of these samples may also be preserved permanently. In environmental research projects, it is prudent that contextual data of reference and study materials and of observational and sampling events are maintained in a database system or workbench (1).

Scientists may collect samples, when neither having a certain research project context in mind nor a corresponding workflow and data management system. Therefore, there is need of guidance for how to create consistent and unique (meta-) data already in the field, which will allow for identification of the original sampling object with regard to geodata and for how subsequent data processing of the physical and connected digital objects might be achieved.

The sampling of physical objects alongside gathering precise contextual data during field campaign is most challenging for every researcher, particularly under adverse habitat conditions, since multi-tasking skills are required and certain activities may often be time-intensive. Therefore, new strategies that might support effective sampling and allow for easy simultaneous data gathering are required.

Key requirements of such an approach are as follows: (i) Geo-information and other object-related core information should be gathered by using a device during the field campaign. (ii) Basic linkage of machine-generated core information on physical, i.e. sampled, with digital objects (like images) and contextual data (‘metadata’) should be established already during the field campaign (when initiating gathering event and data generation). In consequence, field activities have to be preceded by preparatory work for creating and individually marking material storage units in a way that later digitalization in the field is facilitated. (iii) Software to establish such digital units during the field campaign should work under online and offline conditions. (iv) The primary digital data unit with machine-generated and automatically processed core information about the gathered physical object (sample) should be made universally identifiable. This can be achieved at a very early ‘data life cycle stage’ as the first steps of data generation/data collection [for the term ‘data life cycle’ see Web-links to DataONE and German Federation for Biological Data (GFBio), below]. The generation of a first version of universally identifiable contextual data should be realized latest immediately after completion of a field campaign.

In the following, a new workflow is described, which fulfils these criteria and facilitates professional and effective sampling of vouchers in the field.

## Description of a generic workflow for effective sampling combined with generation of universally identifiable data

The proposed workflow is process-oriented and consists of several timely and logically dependent steps. Steps 1 and 2 have to be achieved before starting a field campaign. Step 3 needs to be performed during the field campaign only. Step 4 concerns image and image data processing in the laboratory or field station. The same applies to Step 5, referring to subsequent sample processing and data management, including the import of data in a project database or workbench.

### Step 1: Generation of universally unique identifiers (UUID version 4) as primary object identifiers

Before starting a field campaign, several arrangements need to be achieved. One of these concerns the determination of the kind of object identifier to be used in the field for marking physical units or samples. The workflow described here relies on the use of universally unique identifiers (UUID/GUID), version 4 (https://en.wikipedia.org/wiki/Universally_unique_identifier), or parts of these randomly generated code strings of 32 digits. Modifications of the method with alternative identifier types and schemata are thinkable options (2), but are not discussed here. The local UUID system (3) is used since the 80ths of the last century as the basic standard for digital object identifiers in computer networks. The UUID in its DCE variant was first specified in 1997 by ‘The Open Group’ (4), and is now subject of basal ISO standards, e.g. ISO/IEC 11578:1996, ISO/IEC 9834-8:2005 and ISO/IEC 9834-8:2014. Routines for generating UUIDs are widely distributed as online and offline versions. Thus, the UUIDs can be autonomously created and printed by any researcher without any involvement of a controlling institution, consortium, project coordination desk or central registry. UUIDs are universally unique and by their use, numbering conflicts are avoided à priori. If accordingly applied, the UUIDs or human-readable parts of these code strings (e.g. the first 8 digits) are particularly appropriate for semi-automatically linking (a) physical units with physical or digital processes and (b) binary data with non-binary data. The use of the UUID system already makes the original (meta-) data, as gained in the field, universally identifiable. The consistent reuse of this identifier within the subsequent data pipelines is in principle possible and most useful. It may stabilize data processing during the whole data life cycle including data analysis processes and the final data publication with persistent identifiers (PIDs) according to the FAIR principles (5, 6; see also Discussion Section).

### Step 2: Preparation of UUID-QR-code-labelled containers for physical units

In the workflow approach described here labels and labelled containers have to be prepared already before a field campaign has been started. Physical units or samples collected during the field campaign for later analysis and short-time or long-term storage need to be linked with a physical representative of an identifier, e.g. a label, in order to mark them and facilitate the later generation of digital sampling records. This is usually achieved by using printed or handwritten labels with a human-readable number or code, which identifies the physical object. The presence of physical representatives of object identifiers during field campaigns is essential in coupling the workflow (initiated by sampling a physical unit) with the corresponding data record generation (by creating a digital unit).

In life science research projects, sampling activities are frequently undertaken by more than one researcher over a longer period of time. Such activities may not always be tightly coordinated during the campaigns or later. This is considered an argument in favour of using random identifiers like UUIDs Version 4 (or parts of UUIDs) and of printing them on a (empty) sample envelope or attaching them to a (empty) sample container before of their usage in the field.

For human-readability, the UUID may be directly printed on the container and/or on a separate label. For enabling machine-readability and image processing as envisaged in Step 4, the UUID may also be represented as QR-code or another type of a 1D or 2D barcode. A number of freely available or accessible online and offline tools for generating QR-codes or other types of barcodes exist. The UUID-labelled carrier type of material (e.g. labelled paper and polythene bags or plastic tubes or boxes) depends on the type and size of the samples and subsamples to be collected during Step 3.

**Figure 1.**
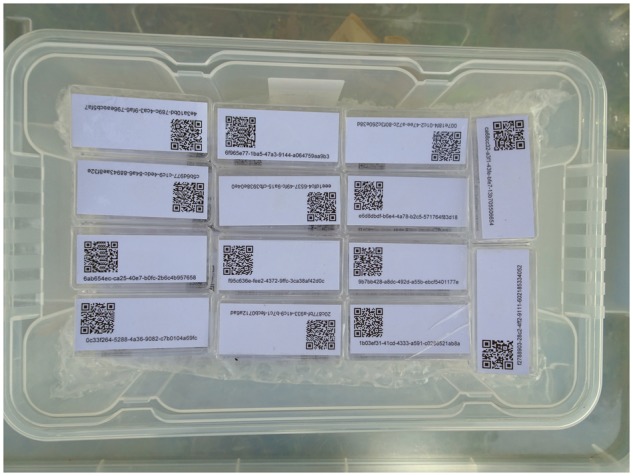
UUID-QR labelled boxes prepared on stock.

Among the easiest collectable and manageable vouchers during field campaigns are, e.g. bryophyte cushions, fungal sporocarps, lichen thalli and soil samples. For managing this kind of objects, paper bags or plastic containers with attached or imprinted UUID-QR-code labels are most appropriate and to be prepared in stock for use during the campaign (Figure 1). Detailed information on Step 2 of the workflow, tools for creating QR-codes and how to manually prepare UUID-QR-coded paper envelopes, useful hints concerning carrier types and their subsequent use of UUID-QR-coding of samples during analysis processes are provided in the Wiki platform of the *Diversity Workbench* (DWB) (http://diversityworkbench.net/Portal/Training_materials).

### Step 3: Sampling and imaging with GNSS- respectively GPS-enabled smartphone

**Figure 2.**
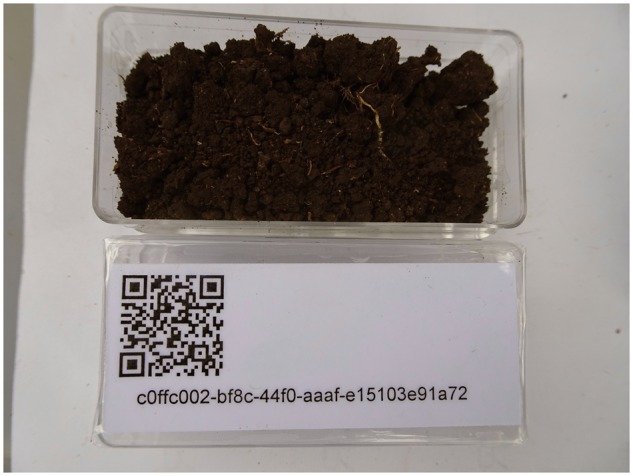
UUID-QR labelled box with soil sample being imaged at the sampling site Ruma (Kenya) with a digital camera with GNSS resp. GPS functionality.

During a field campaign, a high number of sampling events with many accruing samples may take place at a high number of sampling sites. The specimens are usually divided into fitting portions and, for subsequent transport and analysis, put in adequate containers. For assigning an UUID to the samples, the material is placed on or beside the UUID-QR-coded container (as prepared in Step 2) immediately after sampling. Using a GNSS resp. GPS-enabled smartphone with camera (or a digital camera with GPS functionality), one or more images are taken for full documentation of the collection event for one sample (Figure 2). Descriptive information on (handwritten) labels may be attached to the sample and to the container spontaneously or as prepared on stock before the sampling campaign and imaged accordingly. These additional notes might be used as associative elements facilitating a later intuitive sorting of the collected material. The resulting image(s) might comprise the following information: (a) the outer appearance of the sample, optionally, e.g. with additional labels attached providing as image content contextual information or codes as, in case of soil samples, e.g. site, borehole, horizon and replicate numbers; (b) the QR-coded UUID (physical identifier of the sample, subsequently also usable as a digital object identifier) as image content and, (c) the geo-coordinates tag and datetime tag in the Exif image header.

Primary contextual data in life science research are space-time coordinates, i.e. geo-coordinates, altitude and datetime data. They can be recognized as ‘intrinsic metadata,’ being constructed during data capture (5). Information concerning sampling site name and characteristics, or observations, measurements and sampling conditions are supplementary or ‘user-defined.’ Further information that possibly might be assigned in the field to a sampled physical object concerns the taxon name or classification, as well as images of the object and its environment. The digital representative of a physical object or unit (e.g. with taxon name, image or audio file etc.) and the contextual description (e.g. geo-coordinates, datetime and person ID tags) are considered as the minimum or primary information entity for life science data, i.e. part of the metadata (7). The detailed facts on the instance of occurrence of an object, i.e. on the ‘what, where, when, how and by whom’ are imperative. GNSS-enabled smartphones and digital cameras with GNSS functionality provide geo-tagging features and generate as primary keys the camera image file names with camera-specific internal image identifiers. These JPEG and TIFF images include contextual data and IDs in textual format being stored in the Exif image header.

This extractable information is sufficient for later setting up a scientifically valuable digital data unit/record of a taxonomically identified or otherwise categorized or even unclassified object. Thus, the geo-referenced digital images of objects represent a kind of minimum information entity for characterization of observation and sampling records. The digital units (raw data in binary format and textual format) might be processed after completion of a field campaign under Steps 4 and 5.

### Step 4: Image processing with extraction of image content and Exif tag information and data storage

Image processing as described as part of the presented workflow is achieved after completion of a field campaign. It comprises (a) interpretation and extraction of the QR-code information from the image content, (b) extraction of the tag information from the Exif header of the images and combining (a) and (b) into CSV-formatted contextual information and (c) linking images showing one and the same QR-code. During this process, data has to be standardized to some degree. The resulting pre-defined schema with the unit code (UUID) as the new primary key might include, (a) the unit code, (b) date and time, (c) geo-graphic coordinates (preferably in WGS 84 format), (d) altitude (in m asl), (e) author/collector and (f) image files names. Thereby, Step 4 of the workflow results in the generation of identifiable research data.

A suitable software solution for supporting Step 4 would have to provide the following features: (a) reading bulk images for extracting barcode tags and storing such information; (b) extracting and reorganizing Exif header information from core tags; (c) renaming image files (according to naming conventions of a given project) and (d) creating CSV-formatted output text files which contain all the information for import into a data management system. Elements of the extracted UUIDs may be automatedly inserted as a prefix into the original image file name and also be used as primary key.

**Figure 3.**
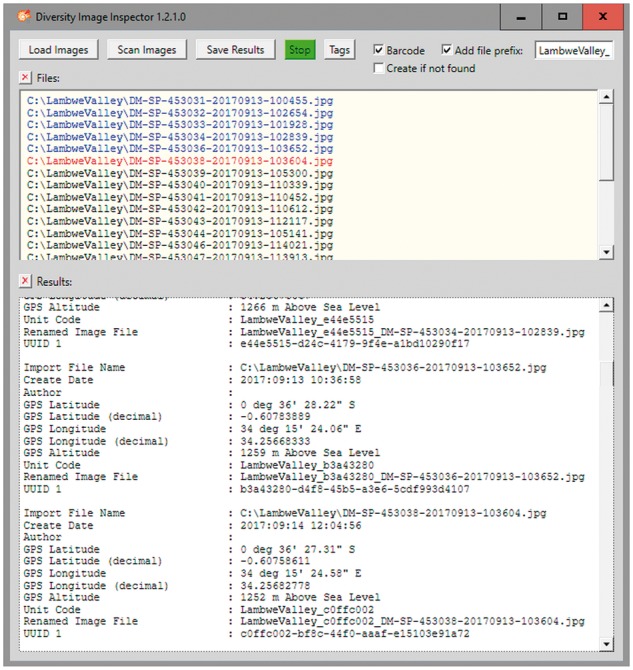
DII user interface. Image files renamed with prefix and the first 8 letters and digits of the version 4 UUID included.

This type of tool has recently been set up as a module of the DWB framework (8). The *DiversityImageInspector* (DII) (http://diversityworkbench.net/Portal/DiversityImageInspector) provides the above-mentioned features and combines functions based on two freeware tools: (a) ExifTool (https://sno.phy.queensu.ca/~phil/exiftool/), for extracting the geo-coordinates and time tags from the image metadata header and (b) ZBar (http://zbar.sourceforge.net), for reading and interpreting barcode information within an image. The latter supports barcodes of the following types: EAN-13/UPC-A, UPC-E, EAN-8, Code 128, Code 39, Interleaved 2 of 5 and QR-code. In the workflow described here, the stand-alone application with user interface allows for extracting the UUIDs from the QR-code elements of bulk image files, as well as the geo-coordinates and time tags from the Exif header information and returning the results into a table (Figure 3). The table may be stored as CSV-formatted text file along with the links to the referenced image files. In addition, an option exists to rename the link address of the image files by adding a project prefix according to the naming convention for image storage of a given data project. This feature might also be used to address the image file storage, e.g. on the server of an image repository.

### Step 5: Subsequent sample processing and data processing 

After completion of the field campaign and of the image processing done under Step 4, the samples and subsamples in the UUID-QR-coded containers need to be processed, curated and stored. They may be subjected to an array of scientific operational steps comprising microscopy, DNA extraction, PCR and DNA sequencing among other methods, depending on the nature of the samples and the specific scientific questions. Some arrangement of the material might be achieved by using the CSV table spreadsheet created under Step 4. The originally assigned UUID (or a truncated version with the first 8 digits) may have to be attached to the samples in the laboratory to link the physical units with the generated geo-referenced sample records.

**Figure 4.**
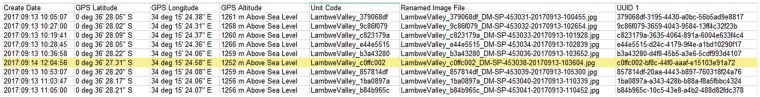
DII export in CSV-formatted text with GNSS coordinates and UUID.

The CSV-formatted text with UUID, GNSS coordinates, time stamp (Figure 4) and links to the image files as result of Step 4 is the starting point for subsequent data management of the (meta-) data using UUIDs as PID.

Most researchers in environmental and ecological sciences tend to use spreadsheets for the management of this kind of tabular life science data together with later assigned other data elements (7). They avoid transforming the data in more sustainable formats and management systems. Thus, the loss of information, e.g. on the interlinkage between data and image objects, is rather predictable. To ensure an appropriate management of the information gained under Step 3 and 4 it is highly recommended to maintain the digital data (with the UUID as identifier) in a well-documented database application designed following community standards for further processing, quality control and analysis.

**Figure 5.**
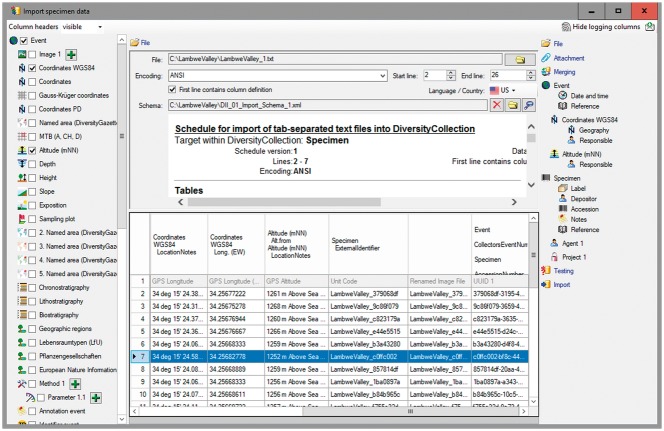
DC Import Wizard with information exported from DII.

**Figure 6.**
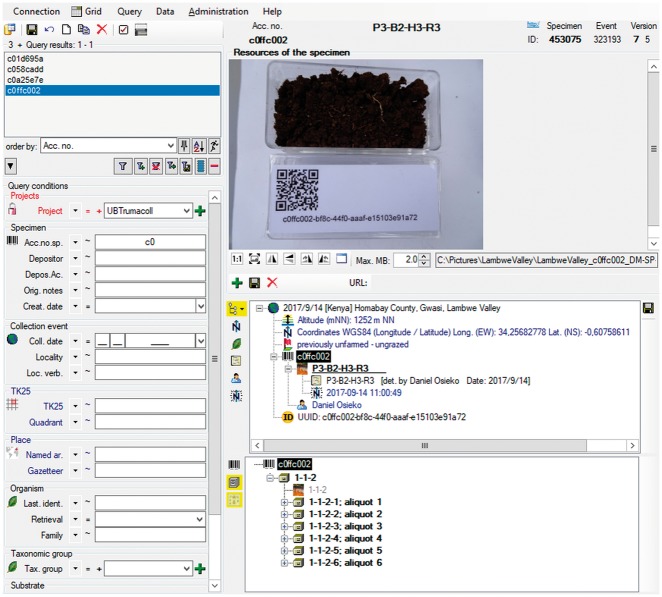
Data record in the relational database DC: objects identifier, information on occurrence, data processing and link to object images.

*DiversityCollection* (DC) (http://diversityworkbench.net/Portal/DiversityCollection) is such a database application based on MS SQL Server as relational database management system (RDBMS) (http://diversityworkbench.net/Portal/Technical_documentation_at_a_glance) and a major component of the DWB platform (8). It supports the management and storage of observational and collection data, and provides an ‘import wizard’ for reading and transforming tabular data to be imported into the corresponding fields of the database. A reference schema with recommended core elements facilitates the import of standard DII export files into DC. It has been published in the SNSB DWB Services GitHub code repository (https://github.com/SNSB/DWB-Contrib/tree/master/DiversityCollection/Import/Schemas/DiversityImageInspector). Additional elements in the DII export table (CSV-formatted text) might also be mapped to DC concepts and imported in DC (Figures 5 and 6). DC comprises >540 concept elements and >50 tables, documented in a name space schema (http://diversityworkbench.net/Portal/dwbCollection) and ER diagram (9). It should, however, be mentioned that there are a number of different database solutions being appropriate for subsequent processing of data from the DII export table.

## Discussion

### Sampling and data maintenance

**Figure 7.**
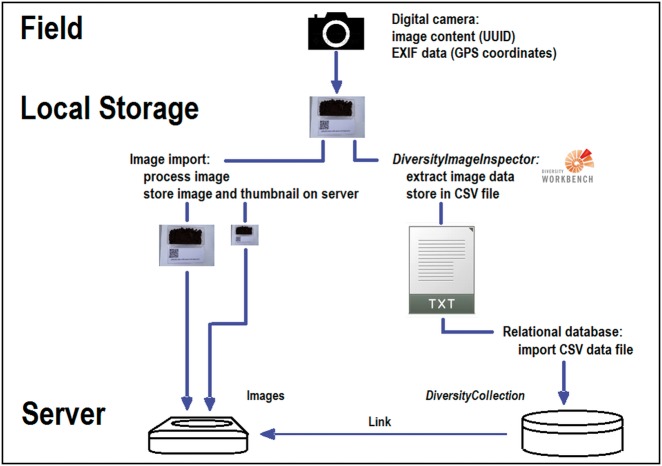
Simplified dataflow from digital camera to CSV document via DII with image storage and data import into the database DC.

Over 3 years, the sketched workflow (Figure 7) as well as the mentioned procedures has been tested with regard to their general utility and usability during various biological field excursions as well as in field courses with MSc life science students. The installation of the newly implemented DWB tool DII on a PC or personal notebook, or similar device is one of the mandatory pre-requisites for the inclusion of the suggested workflow into ongoing monitoring or field research projects. Furthermore, the installation of DC in a minimum configuration or an equivalent database with corresponding import functions is highly recommended to keep the (meta-)data elements connected and the data persistent. It should also be pointed out that data maintenance in advanced management systems like RDBMS, whether accessible via intranet or internet, should start as early as possible during the data life cycle, if possible already during data generation after the return from the field campaign. The DWB applications are appropriate for maintaining many types of biological and environmental data and assigned local identifiers or web-based identifiers. By their usage, the handling of the primary data elements and binary data objects as well as the handling of the connected experiment and analysis data can be organized effectively.

### Data processing

Monitoring and inventorying concerted activities with high-throughput ambitions entail the need of efficient work- and dataflows. The use of ready-made UUID-barcoded containers in the field allows for neglecting registration and synchronization of identifiers among the various subprojects or campaigns. Imaging of UUID-QR-code-labelled containers, using GNSS-enabled smartphone with camera (or a digital camera with GNSS functionality) is mandatory. Even ‘orphan’ samples having been unprocessed for some time, may be, as far as the digital images comprising the contextual information are still available, referable to geo-coordinates and time tags, and thus assignable to a spatial and temporal context. The presented, almost generic data pipeline and workflow with randomly generated local UUIDs, combined with image processing, is therefore considered a step forward to efficient authority-independent, semi-automated gathering and data harvesting in the field.

In general, object identifiers might be either local IDs or web-based, but should be in any case stable and persistent for the object concerned. They are assigned for various purposes, e.g. for organizing a working process, for documentation of storage or institutional inventory, for managing business or administrative actions etc. Identifiers could be categorized either as machine- or human-readable, and are either primarily designed for digital or non-digital objects. Nowadays, web-based object identifiers are gaining rapidly increasing importance in the online publication of data objects and in the world of linked open science data (2, 10).

However, the use of the oldest standard, the local UUID identifiers, as proposed here, has several crucial advantages. If retained in downstream systems (2), it supports the stable and persistent identification of objects already from the point of data generation. Local UUID identifiers are appropriate (a) for physical objects and their derivatives and (b) for identifying and cross-linking digital objects (binary and non-binary) and (c) for data pipelines or workflows in this context. In general, they play an important role to create a stable interconnection between the world of local application networks and internet databases. Furthermore, UUIDs, version 4 or parts thereof could be later in the workflow included as descriptive part of any web-based identifier system and Uniform Resource Name (URN) string (2, Table 1); see also implication for future projects explained at the end of the paper.

In the near future, environmental monitoring projects will probably include mobile machines to a considerable extent, fulfilling robotic-based environmental sampling procedures (11). The proposed workflow or modifications of it (with image processing, assignment of UUIDs and geo-tagging) might also be supportive for such robotic approaches.

### Sample deposition and data publication

Resulting data might be maintained and processed (including the addition of further descriptive and meaningful project identifiers) in the local virtual research environments of workings groups and research groups for a long time. For being published, data has to be transferred to and between recognized data centres and open data repositories. In parallel, the physical objects are stored in recognized institutional Natural Science Collections. These two transactions may take place timely independent from each other and are regularly accompanied each by the creation of new object identifiers, whether web-based or not (2, 10). This data and material transfer might be guided by brokering initiatives like the GFBio for biological research data.

At this critical point with transfer of ownership and storage of data, of images and of physical objects the archiving institutions with their institutional or domain-specific data repositories have to prevent that the link between the formerly assigned identifiers and the newly assigned (institutional) identifiers, i.e. inventory codes, gets disconnected. The same applies to data publishers, who need to document the complete unbroken chain of data provenance when publishing reusable data sets. Thus, the locally assigned UUIDs or parts thereof assigned to physical objects and environmental samples by the researcher and data producer should not to be confused with object numbers or specimen barcodes as assigned to physical object when reference material is transferred to institutional material repositories like internationally recognized Natural Science Collections and biobanks. Such organizations manage long time preservation of voucher specimens and make them available to experts for re-examination. They mostly use in-house standards for assigning accession and inventory numbers as object identifiers to material they own. These standards might also include local UUIDs or web-based identifiers with UUIDs as descriptive parts. Fortunately, most of these institutions keep—beside their standard codes—primary or historical codes assigned to the physical object for documentation with the object, also after the assignment of new inventory or accession numbers. Thus, UUIDs assigned in the field or laboratory will not only stay within the content of the binary data of the images, but probably stay with the material on the long run. Some examples for regular specimen accession numbers or inventory codes are listed in iDigBio (https://www.idigbio.org/wiki/index.php/Specimen_Barcode_and_Labeling_Guide).

A CETAF (http://cetaf.org) service description how to publish inventory codes of Taxonomic Facilities as part of actionable, long-term stable and semantic web compatible identifiers for the distributed access to biological collection objects has recently been published (12). Alternative approaches with central registries are those of SESAR providing an International Geo Sample Number (http://www.geosamples.org) and the case study of MuseumID on providing a Museum Object ID (http://museumid.net/documentation). In all the approaches of contributing to a biodiversity data network relying on megascience platforms (13), the primary identifier created at the point of data generation, here the local UUID, should be part of the core elements published. In earlier non-digital times, the collector name with collecting number played a similar role but without ensuring (global) uniqueness.

The assignment of UUIDs to digital objects very early in the data life cycle has clear advantages: It might be used as primary or secondary key in the data pipelines as later established in the virtual research environments of research groups (e.g. with laboratories and data processing and analysis pipelines there). These data pipelines might proceed independently from the various processing steps of the physical specimens with labels attached with identical UUIDs. Later, the digital representation of the UUIDs might become part of the reference in data publications without direct link and relationship to the related physical objects and their descendents. In such rather frequent cases, UUIDs as applied in the described workflow are considered to be most useful for secondarily linking published data with the physical material stored (with deviating inventory codes) in biobanks or museums.

Services to preserve and publish biological data with all related identifiers assigned during the data life cycle (here the UUIDs) and the direct link to the storage information of physical reference material are currently established by some data centres of the evolving national infrastructure ‘GFBio’ (1) and will gain profit from the approach published here.

Summarizing, the workflow described here has a great potential to improve monitoring and ecological research in the digital age and to cross-link data pipelines across distributed local networks and between local networks and basic internet services. Eighty percentage of the available scientific data objects (digital datasets) are re-useless (6), which means that they have neither been assigned with a PID, nor have intrinsic metadata assigned, nor user-defined provenance data. Such type of data objects might have structured data (elements) or not. In both cases, they fulfil none of the guiding principles of FAIR data (5) and are useless for future research studies. In the case of gathering physical objects in the field with precise geodata, where the assigned data should be persistently and uniquely referring to these primary physical objects, the situation is even worse. The unique relation of the observation and gathering event with the collected physical object to the assigned data is often disconnected already during the field campaign. The approach described here, addresses these problems and supports researchers in producing and publishing valuable geo-referenced data.

Environmental monitoring projects might even think about how to extend the workflow described here and organize the UUIDs as part of web-based identifiers, like URIs (3, 14). This might be achieved by using an URN, e.g. in names schemata as urn: uuid: 8139d48b-f83f-49 cc-81c6-b037f0bc8e6c or as Uniform Resource Locator (URL) like http://mod-co.net/8139d48b-f83f-49cc-81c6-b037f0bc8e6c with the UUID as html page head element ‘title’ and could thus promote the quick representation of geo-referenced data apart from any institutional registration. In the URL case, the project group might register a domain name in the Domain Name System (DNS) and use information in the DNS database together with external generic resolving systems of the internet [(15), and references cited there].

## Web links and abbreviations (alphabetical)

CETAF—Consortium of European Taxonomic Facilities: http://cetaf.org (8 December 2017, date last accessed).

DataONE—Data life cycle: https://www.dataone.org/data-life-cycle (8 December 2017, date last accessed).

DataONE—Data Observation Network for Earth: https://www.dataone.org (8 December 2017, date last accessed).

DiversityCollection: http://diversityworkbench.net/Portal/DiversityCollection (8 December 2017, date last accessed).

DiversityCollection—Name space schema [http://diversityworkbench.net/Portal/dwbCollection (8 December 2017, date last accessed)].

DiversityImageInspector: http://diversityworkbench.net/Portal/DiversityImageInspector (8 December 2017, date last accessed).

DiversityImageInspector—Manual: http://diversityworkbench.net/w/media/9/92/DiversityImageInspectorHelp_1_2_1_0.pdf (8 December 2017, date last accessed).

Diversity Workbench—Technical documentation at a glance: http://diversityworkbench.net/Portal/Technical_documentation_at_a_glance (8 December 2017, date last accessed).

Diversity Workbench—Training Materials: http://diversityworkbench.net/Portal/Training_materials (8 December 2017, date last accessed).

ExifTool: https://sno.phy.queensu.ca/~phil/exiftool/ (8 December 2017, date last accessed).

GFBio—Data life cycle: https://www.gfbio.org/training/materials/data-lifecycle (8 December 2017, date last accessed).

GFBio—German Federation for Biological Data: https://www.gfbio.org (8 December 2017, date last accessed).

GNSS—Global Navigation Satellite System: https://en.wikipedia.org/wiki/GNSS_applications (8 December 2017, date last accessed).

iDigBio—Specimen Barcode and Labeling Guide: https://www.idigbio.org/wiki/index.php/Specimen_Barcode_and_Labeling_Guide (8 December 2017, date last accessed).

MOD-CO—Meta-omics data of collection objects: http://www.mod-co.net (8 December 2017, date last accessed).

MuseumID—Identifying museums and museum objects on the internet: http://museumid.net/documentation (8 December 2017, date last accessed).

SESAR—The System for Earth Sample Registration: http://www.geosamples.org (8 December 2017, date last accessed).

SNSB DWB Services GitHub code repository—DiversityImageInspector: https://github.com/SNSB/DWB-Contrib/tree/master/DiversityCollection/Import/Schemas/DiversityImageInspector (8 December 2017, date last accessed).

UUID—Universally unique identifier: https://en.wikipedia.org/wiki/Universally_unique_identifier (8 December 2017, date last accessed).

ZBar—ZBar barcode reader: http://zbar.sourceforge.net (8 December 2017, date last accessed).
